# Cross-language activation of morphological relatives in cognates: the role of orthographic overlap and task-related processing

**DOI:** 10.3389/fnhum.2015.00016

**Published:** 2015-02-02

**Authors:** Kimberley Mulder, Ton Dijkstra, R. Harald Baayen

**Affiliations:** ^1^Centre for Language Studies, Radboud University NijmegenNijmegen, Netherlands; ^2^Donders Institute for Brain, Cognition, and Behaviour, Radboud University NijmegenNijmegen, Netherlands; ^3^Department of Linguistics, Eberhard Karls UniversityTübingen, Germany

**Keywords:** morphological family size, bilingual word recognition, response competition, cognates, spreading activation

## Abstract

We considered the role of orthography and task-related processing mechanisms in the activation of morphologically related complex words during bilingual word processing. So far, it has only been shown that such morphologically related words (i.e., morphological family members) are activated through the semantic and morphological overlap they share with the target word. In this study, we investigated family size effects in Dutch-English identical cognates (e.g., *tent* in both languages), non-identical cognates (e.g., *pil* and *pill*, in English and Dutch, respectively), and non-cognates (e.g., *chicken* in English). Because of their cross-linguistic overlap in orthography, reading a cognate can result in activation of family members both languages. Cognates are therefore well-suited for studying mechanisms underlying bilingual activation of morphologically complex words. We investigated family size effects in an English lexical decision task and a Dutch-English language decision task, both performed by Dutch-English bilinguals. English lexical decision showed a facilitatory effect of English and Dutch family size on the processing of English-Dutch cognates relative to English non-cognates. These family size effects were not dependent on cognate type. In contrast, for language decision, in which a bilingual context is created, Dutch and English family size effects were inhibitory. Here, the combined family size of both languages turned out to better predict reaction time than the separate family size in Dutch or English. Moreover, the combined family size interacted with cognate type: the response to identical cognates was slowed by morphological family members in both languages. We conclude that (1) family size effects are sensitive to the task performed on the lexical items, and (2) depend on both semantic and formal aspects of bilingual word processing. We discuss various mechanisms that can explain the observed family size effects in a spreading activation framework.

## INTRODUCTION

Past research has shown that the mental lexicon is a highly interactive system, in which words that share orthographic/phonological, morphological, or semantic features can be co-activated along with the actually presented word. One demonstration of this interactive nature is the finding that upon reading a word like *house*, morphologically related complex words are co-activated that contain this word, like *housekeeper, housing,* and *wheelhouse* (Schreuder and Baayen, 1997). The set of activated words has been called the ‘morphological family’ of the target word. Even more intriguing, reading the same English word *house* may activate morphologically related complex Dutch words such as *bejaardenhuis* ‘elderly home’ or *huizenmarkt* ‘house market’ in speakers that are familiar with both of these languages (Mulder et al., 2013). The set of Dutch items that is morphologically related to the English target words is called the ‘cross-language morphological family’ of that word.

This paper provides a more detailed investigation into the activation of such morphologically related complex words in bilingual word processing. More specifically, we investigate how the word recognition of bilinguals is affected by the activation of morphological families from one or both of their languages. These effects of within-language and cross-language family size (i.e., the total number of morphological family members of a word in the same or another language) are investigated in two different paradigms (lexical decision and language decision) and for three different types of words (identical and non-identical cognates, and non-cognates). The manipulation of task and item type allows us to test the hypothesis that bilingual family size effects vary in accordance with task demands and degree of cross-linguistic orthographic overlap. This extends current theories of morphological family size effects that have been proposed for monolinguals, and allows the development of a bilingual model for such effects. To set the stage for our experiments, we will first discuss the nature of family size effects in monolinguals and then address possible implications for bilingual processing.

In monolingual studies, words with larger morphological families are generally found to be processed faster and more accurately than words with smaller morphological families. Facilitatory effects are observed in lexical decision studies for several languages with a concatenative morphology (e.g., for Dutch: Schreuder and Baayen, 1997; Bertram et al., 2000; De Jong et al., 2000; De Jong, 2002; Kuperman et al., 2009; for English: Baayen et al., 1997; De Jong et al., 2002; Juhasz and Berkowitz, 2011; for (non-Germanic) Finnish: Moscoso del Prado Martín et al., 2004; Kuperman et al., 2008). Moreover, facilitatory effects are also observed for languages with an alphabetic writing system and a non-concatenative morphology (for Hebrew: Moscoso del Prado Martín et al., 2005; for Arabic: Boudelaa and Marslen-Wilson, 2011). Finally, written Chinese is non-alphabetic and non-concatenative, but shows effects similar to the family size effect in terms of the productivity of semantic radicals (Feldman and Siok, 1997).

Schreuder and Baayen (1997) explained facilitatory family size effects by means of global lexical activation along the lines of the multiple read-out model of Grainger and Jacobs (1996): words that co-activate many other words (lemmas^1^) give rise to more global lexical activation supporting a positive lexicality decision. De Jong et al. (2003) simulated this mechanism in a computational model of monolingual morphological processing the Morphological Family Resonance Model (MFRM). They showed that read-out of global activation may not be necessary if activation is allowed to resonate between forms, lemmas, and meanings. In their model, associated lemmas (family members) of a target word are activated via the semantic representation of that target word, but not via its form representation. When a semantic representation of a target word is linked to many associated lemmas, a large amount of activation is spread back and forth between this semantic representation and the associated lemmas, gradually increasing the shared semantic activation and the activation level of the target lemma. Such resonance within the morphological family will thus speed up the rate at which the activation of the target lemma increases, resulting in faster word recognition.

Thus, although morphological family members are connected to a target word via both orthographic and semantic links, family size effects are generally assumed to be semantically driven (e.g., Schreuder and Baayen, 1997; Bertram et al., 2000; De Jong et al., 2000; Moscoso del Prado Martín et al., 2005; Mulder et al., 2013, 2014). For instance, Schreuder and Baayen (1997) showed that only semantically transparent family members contribute to the family size effect. Moscoso del Prado Martín et al. (2005) even observed inhibitory family size effects for family members that were not semantically related to the target. In line with this, words with a large family size (the family members being semantically related to the target; Mulder et al., 2013) and words with a large number of orthographic neighbors (the neighbors being semantically unrelated; Holcomb et al., 2002; Müller et al., 2010) elicited different N400 effects, showing facilitation and inhibitory effects on word processing, respectively. Finally, Mulder et al. (2014) investigated primary and secondary family size effects. Secondary family size concerns the number of family members of family members. As an example, *work force* is a secondary family member of *clock*, because it is morphologically related to *clock work*, which is a primary family member of *clock*. Facilitatory effects of English primary family size were observed, while the activation of secondary family members elicited inhibitory effects, showing that when activation spreads too far out, to words that are semantically unrelated to the target, word processing is hindered. In sum, when these various observations are combined, they give rise to the hypothesis that it is the semantic convergence or divergence between target word and family members that determines the direction of the family size effect.

Although these studies indicate that the family size effect is predominantly semantic in nature, the role of orthography in activating morphologically related complex words has never been sufficiently investigated. In a lexical decision task with Dutch monolinguals, De Jong et al. (2000) observed family size effects for both regular and irregular past participles (e.g., *roei – geroeid*, ‘row – rowed’ versus *vecht – gevochten,* ‘fight – fought’), even though the irregular past participle did not share the exact orthographic form with its stem and other family members. This suggests that, at least in monolinguals, the activation of morphologically related complex words is not dependent on complete orthographic overlap between target word and family members.

In the present paper, we investigate whether this finding can be generalized to family size effects in bilingual word processing and across empirical tasks. In bilingual processing, the co-activation of words in the non-target language for a large part depends on the degree of formal overlap between words in these languages. There are word pairs that have complete orthographic and nearly complete semantic overlap across languages. Such words are called identical cognates. An example word pair is provided by the English and Dutch word *tent*. Other translation equivalents have form overlap, but it is partial. An example word pair is *pill* (English) – *pil* (Dutch). Finally, there are translation equivalents with little or no overlap, for instance *chicken* (English) – *kip* (Dutch). In word processing by Dutch-English bilinguals, the English word *tent* is more likely to activate the Dutch orthographically similar word *tent* than *chicken* is to activate its Dutch translation equivalent *kip*. In the present paper, we hypothesize that differences in cross-linguistic overlap will have consequences for the activation of morphological family members in the two languages. Said differently, we expect that morphological family size effects will differ between identical cognates, non-identical cognates, and non-cognates. If this is the case, morphological family size effects are shown to be sensitive not only to cross-linguistic semantic overlap, but also to orthographic aspects of input words.

Until now, the few studies that addressed family size effects in bilingual word processing did not pay attention to this aspect. Some studies only used items that had complete orthographic overlap but different meanings between languages, i.e., interlingual homographs such as the English and Dutch word *room*, meaning ‘cream’ in Dutch; Dijkstra et al., 2005). Other studies did vary the degree of cross-linguistic orthographic overlap, but they did not consider how family size effects depended on such orthographic overlap between word representations (i.e., family size in Dutch-English cognates such as *tent/tent* and *pill/pil* in Mulder et al., 2013). In this paper, we test the effects of orthographic overlap by examining cross-language family effects in both identical (e.g., the English-Dutch word *tent*) and non-identical cognates (e.g., *admiral* – *admiraal*, in English and Dutch). Cognates are particularly useful to examine cross-language effects of family size, not only because their degree of orthographic overlap can be manipulated, but also because they can reveal whether, despite their overlap in semantics, the activation of cross-language family members facilitate a response in different task contexts.

We can extend the predictions of the monolingual MFRM, mentioned above, to bilingual family effects. The model suggests that the cross-language family size effect should be predominantly based on semantic co-activation and resonance between the semantic representation of the target word and the family members. Therefore, regardless of task, the response to a cognate would always be facilitated, because any converging cross-language semantic information strengthens the activation of the target. However, if family members are activated initially in a ‘bottom-up’ way via orthography, cross-language family size effects not necessarily facilitatory, because they may induce response competition between activated within-language and cross-language representations. Moreover, if family members are activated via orthography, then the activation of cross-language family members depends on the degree of orthographic overlap between cognate representations. This means that upon reading an English target word like *work*, the Dutch family member *werkvergunning’* ‘work permit’ is activated to a lesser extent than the English family member *workspace* as a result of less orthographic overlap. In this case, effects of family size should then interact with cognate type.

We further investigate to what extent the effects of cross-linguistic orthographic overlap are task sensitive. To do so, we examine how cross-language family size affects the response to two types of cognates (with complete and non-complete form overlap) and non-cognates in a lexical decision task and a language decision task. In English lexical decision (Experiment 1), participants must decide if the input letter string is an English word or not. Because both readings of a cognate will become activated on the basis of the input letter string, a cognate facilitation effect should arise that is dependent on the degree of cross-linguistic orthographic overlap (thus, it will be larger for identical cognates than for non-identical cognates). Given the demands of the task, participants should base their response read-out primarily on the English lexical representation and English language membership of the word (Dijkstra, 2007). There will be relatively little time for the Dutch orthographic reading of the cognate to activate its family members; as a result, the activation of cross-language family members is expected to proceed indirectly and especially via semantic co-activation. This should lead to facilitatory family size effects for both identical and non-identical cognates, with relatively little difference between both types.

In contrast, in English-Dutch language decision (Experiment 2), participants have to decide as quickly and accurately as possible whether a presented letter string is an English word or a Dutch word. In the case of a cognate, a response conflict is expected to arise, because of the formal overlap between cognate representations. For instance, the words *tent* and *admiral* – in Dutch ‘*admiraal’* – could activate both a Dutch and English response. As a consequence, the response competition between the two readings of a cognate should result in a cognate inhibition effect (cf. Dijkstra et al., 2010). In this paradigm, co-activation of cross-language family members might be expected to lead either to facilitatory effects (because both families strengthen the activation of the target word via semantics) or to inhibitory effects (because of response competition and because both families reinforce English and Dutch language nodes). Especially in this mixed-language paradigm, in which the orthography is important for making a correct decision about the language membership of a word, an interaction between family size and cognate type is expected.

In all, we test the hypotheses that morphological family size is sensitive to cross-linguistic overlap and to task demands by including different item types (identical and non-identical cognates, and non-cognates) in two bilingual experiments: English lexical decision (Experiment 1) and English-Dutch language decision (Experiment 2).

## EXPERIMENT 1 – ENGLISH LEXICAL DECISION

### METHOD

#### Participants

Twenty-nine native speakers of Dutch, mainly students of the Radboud University Nijmegen (mean age 23.8 years, SD = 5.49) took part in this experiment. All participants had English as their second language, having learnt English at school from around the age of 11. All had normal or corrected–to-normal vision. Participants were paid or received course credits for participating in the experiment.

#### Materials

The stimulus set consisted of 400 items, half of which were English words and half were pseudo-words. All word items were selected from the CELEX database (Baayen et al., 1995). Only word items with an English lemma frequency of at least one per million in the CELEX lexical database and a length between three and eight letters were selected. All word items were mono-morphemic words. For each item, the English family size values and the English lemma frequencies per million were extracted from the CELEX database and logarithmically transformed. The English morphological family of a word in CELEX consists of the number of English morphological derivations and compounds of a given word (not including inflections; for studies on inflectional family size effects, see Bertram et al., 2000; Traficante and Burani, 2003).

The experimental items were 90 Dutch-English cognates. Forty of these items were identical in form in Dutch and English (identical cognates; e.g., *horizon*–*horizon*), while the other 50 items were nearly identical in orthography in both languages (non-identical cognates; e.g., *admiral*–*admiraal*). The non-identical cognates were always presented in their English form. The degree of orthographical overlap was calculated by the Levenshtein (1966) distance measure. For each cognate item, the Dutch family size values and the Dutch lemma frequencies per million were extracted from the CELEX database and logarithmically transformed. Similar to the English family size values in CELEX, the Dutch morphological family of a word consists of the number of Dutch morphological derivations and compounds of a given word (not including inflections). Half of the identical and half of the non-identical cognates had a large family size in Dutch, while the other half of these cognates had a small Dutch family size. The sets of identical and non-identical cognates with a large Dutch family size were matched on English Frequency, English Family Size^2^ and Length (in letters) to the identical and non-identical cognates with small Dutch family size (*t*-tests, all *p*’s > 0.05). Moreover, the non-identical cognates with large and small family size were matched on Levenshtein Distance.

The experiment further included 90 English non-cognate words that were matched to the set of cognates on English Frequency, English Family Size, and Length, and 20 English filler words that were matched on Length to the cognates and non-cognates. Finally, 200 pseudo-words were added that were matched to the set of 200 word items on Length. These pseudo-words could be orthographically and phonologically legal words in English. **Table 1** presents the characteristics of the cognate and non-cognate items. The order of word and pseudo-word items was then pseudo-randomized with the restriction that no more than four words or pseudo-words were allowed to follow each other. A new pseudo-randomization was made for each participant.

**Table 1:** Item characteristics of the experimental items used in Experiment 1.

	Identical cognates		Non-identical cognates		English non-cognates
	Large family size	Small family size	Large family size	Small family size	
Length	4.6	5.1	4.92	5.08	4.99
Levenshtein distance	0	0	1.48	1.28	-
Log English frequency	3.53	3.62	3.51	3.14	3.44
Log English family size	2.22	1.77	1.82	1.86	1.82
Log Dutch frequency	3.45	2.99	3.33	2.90	-
Log Dutch family size	**3.49**	**0.92**	**3.65**	**1.19**	-

#### Procedure

Participants performed an English visual lexical decision task. In this task, participants decide whether or not the visually presented stimulus is an existing English word by pressing a button corresponding to either the answer ‘yes’ or ‘no.’ The task was developed and carried out in *Presentation* version 13.0 (Neurobehavioral Systems^3^) and was run on a HP Compaq Intel Core 2 computer with 1.58 GHz memory and a refresh rate of 120 Hz. The participants were seated at a table at a 60 cm distance from the computer screen. The visual stimuli were presented in white capital letters (24 points) in font Arial in the middle of the screen on a dark gray background. Participants were tested individually in a soundproof room. The study was approved by the ethical committee of the Faculty of Social Sciences at Radboud University (ECG2912-2711-059).

Participants first read the English instructions, which informed them that they would be presented with word strings and which asked them to push the ‘yes’ button if the letter string they saw was an existing English word and to push the ‘no’ button if it was not. They were asked to react as accurately and quickly as possible. Participants pushed the ‘yes’ button with the index finger of their dominant hand and the ‘no’ response with the index finger of their non-dominant hand.

Each trial started with the presentation of a black fixation point ‘+,’ which was displayed in the middle of the screen for 700 ms. After 300 ms the target stimulus was presented. The stimulus disappeared when the participant pressed a button, or when a time limit of 1500 ms was reached, and a new trial was started after an empty black screen of 500 ms.

The experiment was divided in two parts of equal length. The first part was preceded by 20 practice trials. After the practice trials, the participant could ask questions before continuing with the experimental trials. The two parts each contained 200 experimental trials. The proportion of items from each condition was the same in the two parts of the experiment. Each part began with three dummy trials to avoid lack of attention during the beginning of the two parts. The end of the first part was indicated by a pause screen. The experiment lasted for approximately 16 minutes.

After completing the lexical decision task, participants performed the X-LEX (Meara and Milton, 2003). This task was used to obtain a general indication of their proficiency in English in terms of vocabulary knowledge. Based on their scores (all scores >3200), all participants could be qualified as highly or intermediately proficient in English. Finally, participants were asked to fill out a language background questionnaire. The total session lasted approximately 30 minutes.

### RESULTS

Data cleaning was first carried out based on the error rate for participants and word items. Participants with an error rate of more than 15% on the word items were removed from the data set (participant accuracy mean ranged from 66 to 99%), which resulted in the exclusion of the data from five participants.

Three word items (*lung, alley,* and* toad*) that elicited errors in more than 25% of the trials were removed from the data set. After removal of these items, we were left with 4243 data points on the word items. RTs from incorrect responses or null responses were removed from the remaining data set (4.18% of the data points). This resulted in a data set with 4058 data points. Inspection of the distribution of the response latencies revealed non-normality. A comparison of a log transform and an inverse transform (RT = 1000/RT) revealed that the inverse transform was most successful in approximating this non-normality.

Response latencies were analyzed with a linear mixed effects model with subject and item as crossed random effects (see, e.g., Baayen, 2008; Baayen et al., 2008). We considered the following predictors: one lexical variable that is known to affect response latencies is target word frequency. Recent research shows that *SUBTLWF* (logarithmical transformation of English Subtitle frequency per million) is a better predictor of response latencies than the logarithmically transformed English CELEX frequencies per million (see Brysbaert and New, 2009). In the remainder of this experiment, we will use the term *English Frequency* to refer to the logarithmical transformation of *SUBTLWF* as a predictor of target word frequency. Moreover, because bilinguals are expected to be sensitive to non-target language word frequency, we considered the logarithmically transformed CELEX values per million for Dutch lemma frequency (*Dutch Frequency*).

Further, the logarithmically transformed CELEX values for English family size (*English Family Size*) and Dutch family size (*Dutch Family Size*) were included as predictors. The English family size values were collinear with the values of the logarithmically transformed values of *English Frequency* and *Dutch Family Size*. To remove collinearity, we regressed *English Family Size* on *English Frequency* and *Dutch Family Size* and used the resulting residuals as new predictors of English family size uncontaminated by English frequency. Similarly, *Dutch Family Size* was regressed on *Dutch Frequency* and *English Family Size*. Moreover, we added the predictor *Total Family Size* (the sum of the Dutch and English family sizes) to account for possible increased facilitation due to large amount of global activation in the lexicon produced by the family members.

Besides these predictors for target and non-target language family size and frequency, other predictors were considered that could affect lexical decision latencies. In order to test whether cognate items were processed differently from non-cognate items, we included a factor *Cognate* with the levels ‘cognate’ and ‘non-cognate.’ Moreover, the predictor *Word type*, containing three levels (‘identical cognate,’ non-identical-cognate,’ and ‘non-cognates’), was included to account for the degree of form overlap between English and Dutch, with non-cognates having zero overlap, non-identical cognates having intermediate overlap, and identical cognates having maximal overlap. Furthermore, to be able to account for the possibility that family size effects are dependent on a “complete-or-not-complete” distinction in formal overlap, the factor *Identical Cognate* [with the levels Identical cognates and Other items (the latter including non-identical cognates and non-cognates)] was considered.

Further, *OLD* (the mean distance, in number of steps, from a word to the 20 closest Levenshtein neighbors in the lexicon; OLD-20; see Balota et al., 2007, and Yarkoni et al., 2008) was included as a predictor to account for effects of similarity between English words. Finally, we included *Trial* (the rank of the item in the experimental list) as predictor to account for learning effects during the experiment.

We performed a stepwise variable selection procedure in which non-significant predictors were removed to obtain the most parsimonious model. Moreover, for each significant predictor, it was evaluated whether inclusion of this predictor resulted in a better model (i.e., containing a lower AIC compared to when this predictor was not part of the model). Next, potentially harmful outliers (defined as data points with standardized residuals exceeding 2.5 standard deviation units) were removed from the data set. We then fitted a new model with the same significant predictors to this trimmed data set.

**FIGURE 1:**
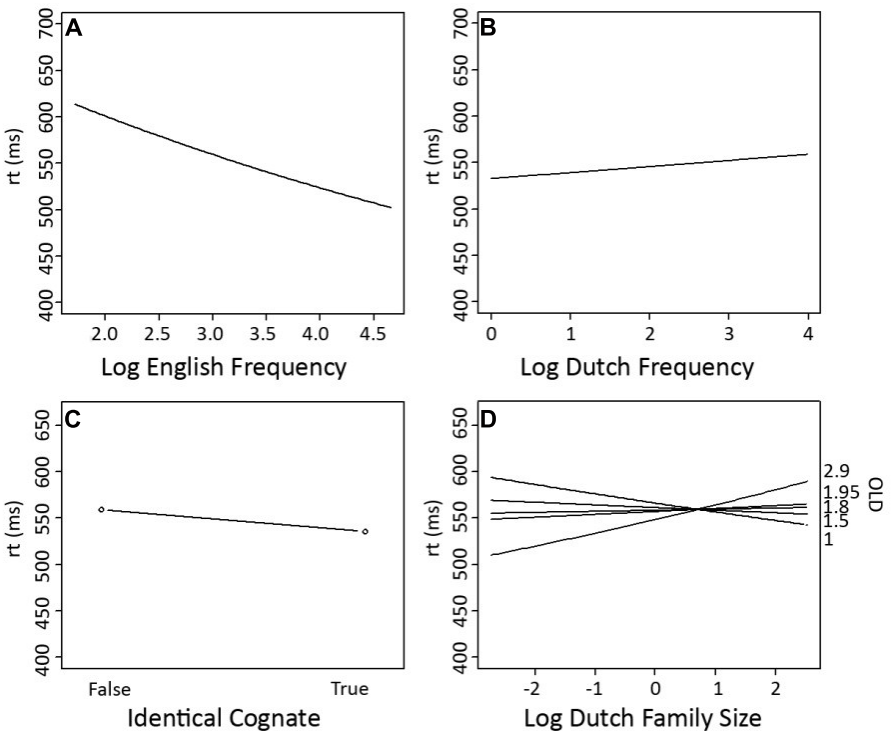
**Partial effects of the significant predictors on response latencies in English lexical decision (Experiment 1). (A)** Log English Frequency, **(B)** Log Dutch Family Size, **(C)** Identical Cognate (2 levels: True, corresponding to the set of identical cognates, and False, corresponding to the set of non-identical cognates and non-cognates), and **(D)** The interaction of Log Dutch Family Size with OLD.

The final model incorporated three parameters for the random-effects structure of the data: a standard deviation for the random intercepts for subject (SD = 0.21) and item (SD = 0.08), as well as a SD for the by-subject random slope for *Trial* (SD = 0.05). The standard deviation for residual error was 0.29. The model contained four numerical predictors (*English Frequency, Dutch Frequency*, *Dutch Family Size*, and *OLD*), one factorial predictor (*Identical Cognate*) and one two-way interaction (*Dutch Family Size*: O*LD*). The relevant statistics and corresponding coefficients of the final model are reported in **Table 2**. The significant partial effects of the final model are visualized in **Figure 1**. In both **Table 2** and **Figure 1C**, the two levels of *Identical Cognate* are specified as *True* and *False*: the former corresponding to the set of identical cognates, and the latter to the set of non-identical cognates and non-cognates.

**Table 2:** Coefficients of the main effects and interaction effects of the final model, together with the standard error, *t*-values and *p*-values in English lexical decision (Experiment 1).

	Estimate	SE	*t*-value	*p*-value
Intercept	-1.454	0.084	-17.279	0.000
English frequency	-0.123	0.017	-7.409	0.000
Dutch frequency	0.022	0.010	2.149	0.002
Dutch family size	-0.073	0.039	-1.857	0.048
OLD	-0.029	0.018	-1.629	0.087
Identical cognate *true*	-0.078	0.021	-3.715	0.000
Dutch family size: OLD	0.043	0.019	2.202	0.019

The analyses showed a facilitatory effect on response latencies for *English Frequency*, while (non-target language) *Dutch Frequency* had an inhibitory effect. Moreover, the final model revealed a processing advantage for identical cognates in comparison to non-identical cognates and non-cognates. While models including either the predictors *Cognate* or *Word Type* also produced significant facilitation effects for cognates in comparison to non-cognates, with the latter predictor indicating the largest facilitation effects for identical cognates, *Identical Cognate* turned out to be a better predictor than either *Cognate* or* Word Type*, suggesting that it is maximal formal overlap with Dutch words that is most helpful in order to make an L2 lexical decision.

*Dutch Family Size* was a better predictor than *Total Family Size,* which was not significant. *Dutch Family Size* has a significant facilitatory main effect on response latency. However, the significant interaction between *Dutch Family Size* and *OLD,* shows that response latencies were slower when a word has a large *Dutch Family Size* and fewer close orthographic neighbors. However, when a word has more close orthographic neighbors, a large *Dutch Family Size* is beneficial to word processing. No significant interaction between *Dutch Family Size* and either *Cognate Type* or *Identical Cognate* was observed.

### DISCUSSION

As predicted, in the English lexical decision task of Experiment 1, Dutch-English bilinguals were sensitive to the frequency of the English target words. English words with a higher frequency led to faster responses than lower frequency words. The effect of *English Family Size* of the target words was not significant. This is not surprising, because this factor was controlled for in order to allow non-target language (Dutch) family size effects to arise.

Importantly, statistical analyses revealed a significant effect of *Identical Cognate*. This predictor turned out to be a better predictor than both *Cognate* and *Word Type*. Responses to identical cognates were faster than to non-identical cognates and non-cognates. This result supports the distinction between identical cognates and non-identical cognates. This dissociation between the two cognate types is in line with the findings of Dijkstra et al. (2010), who observed a gradual decrease in L2 response latencies with an increase in similarity for non-identical cognates and a steep decline in response latencies going from non-identical to identical cognates. As the major mechanism underlying these findings, Dijkstra et al. (2010) proposed that the non-target L1 reading of the presented cognate was activated to an extent dependent on its degree of overlap with the input letter string. This then resulted in differences in semantic co-activation.

There was a significant facilitatory main effect of *Dutch Family Size.* Moreover, *Dutch Family Size* interacted significantly with *OLD*, a measure of orthographic neighborhood density. The interaction revealed a processing disadvantage for words with a large Dutch family size and more distant English orthographic neighbors. Thus, making a lexical decision on an English word is easier when a word is more ‘English-like’ (e.g., when it is orthographically closer to English neighbors) and generates less Dutch activation (e.g., when it has a small Dutch family size).

Interestingly, no significant interaction was observed between *Dutch Family Size* and *Identical Cognate*. A lack of a difference in the direction of the effect or the effect size for identical and non-identical cognates would follow if the family size effect is exclusively semantically driven. Therefore, although a morphological relationship links a target word to its family members, it seems that the effect of the activation of these family members itself is not dependent on the degree of formal overlap they share with the target word. However, while this may be true for the present situation in which bilinguals processed words in a largely monolingual task context, formal overlap might affect the family size effect when there is an explicit bilingual task context. This would especially be the case for a language decision task in which bilinguals have to judge the language membership of presented words (e.g., English or Dutch).

This issue is investigated in Experiment 2. Here Dutch-English bilinguals carried out a Dutch-English language decision task, in which they had to decide whether or not a presented word was English or Dutch. There were no pseudo-words in this task. In this task, the two readings of a cognate are linked to a different response. For instance, in Dutch-English language decision, the English reading *work* of the cognate *work* is linked to an English response, while the Dutch reading *werk* is linked to a Dutch response. Making a language decision on a cognate should therefore result in response competition between the representations of a cognate and slow down target word processing. The task dependency of processing form similar words was earlier observed for both interlingual homographs (Dijkstra et al., 1998, 2000; Dijkstra, 2005) and cognates (Font, 2001; Dijkstra et al., 2010) showing a change in the directionality of the effects in (generalized) lexical decision and language decision. Moreover, Dijkstra et al. (2010) observed a discontinuous strong increase in response latencies in language decision going from nearly identical to identical cognates, mirroring the cognate effects found in lexical decision.

As was hypothesized in the Introduction, the activation of morphological family members of a cognate in language decision may affect target word processing in two ways. First, given that morphological family members of a cognate share part of their semantics with the cognate, activation of both within-language and cross-language family members could lead to facilitation for cognates with a large family size. This will then reduce the cognate inhibition effect.

Alternatively, activated morphological families may inhibit word processing given that they are linked to cognate representations that are in response conflict. Because family members are assumed to strengthen the activation of the target word to which they are linked, cognates with a large family size could then strengthen response competition and increase the cognate inhibition effect. Moreover, if language-specific information is necessary in order to resolve a response conflict, then family size effects might be sensitive to the degree of form overlap between cognate representations. If this is the case, stronger inhibitory effects of the family size of both languages are expected in identical cognates compared to non-identical cognates, because they activate less language-specific information.

## EXPERIMENT 2 – DUTCH-ENGLISH LANGUAGE DECISION

### METHOD

#### Participants

Forty-five students of Radboud University Nijmegen (mean age 20.4 years, SD = 1.92) took part in this experiment. They were all native speakers of Dutch, having English as their second language. They were first exposed to English at school, approximately from the age of 11. They were paid or received course credits for participating in the experiment.

#### Materials

The stimulus set consisted of 168 items. The set consisted of 72 Dutch-English noun cognates and 96 non-cognate items. The 72 cognate items were 24 form-identical Dutch-English cognates and 48 Dutch-English cognates that were not identical in form. The 96 non-cognate items were 48 English non-cognates and 48 Dutch non-cognates.

Because of the change from an English lexical decision task in Experiment 1 to an English-Dutch language decision task in Experiment 2, Dutch non-cognates and non-identical cognates had to be added to the stimulus materials. Further, 20 of the 90 cognates and 20 out of 90 non-cognates that were used in Experiment 1 (lexical decision) were also used in Experiment 2 (language decision). In Experiment 1, in order to observe Dutch family size effects, English family size was controlled for. As we wanted to look at response competition between the Dutch and English and the contribution of their respective family sizes, we had to vary the English and Dutch family sizes; as a consequence, the item set of Experiment 1 was not completely suited for Experiment 2^4^.

The 48 non-identical cognates were either presented in Dutch or English orthography. A participant was presented with only half of the non-identical cognates in their Dutch form and the other half in their English form. Thus, for each participant, half of the items were Dutch and half of the items were English (24 identical cognates, which could be both Dutch and English). In total, there were 72 Dutch words (24 Dutch non-identical cognates and 48 Dutch non-cognates) and 72 English words (24 English non-identical cognates and 48 English non-cognates).

Within each version, the two sets of 24 non-identical cognates were matched to each other on English Family Size and Dutch Family Size, English Frequency and Dutch Frequency (see Experiment 1 for a definition), Length (in letters), log English Bigram Frequency and log Dutch Bigram Frequency. Furthermore, the two sets of 24 language specific non-identical cognates of version 1 were matched on Length and their language specific bigram frequency with the non-identical cognates from the same language in version 2. Finally, the identical cognates were matched on Length, English Frequency, and English Family Size to the set of 48 non-identical cognate items, but could not be matched on Dutch Family Size and Dutch Frequency. The identical cognates have a lower mean Dutch Frequency and are less productive in terms of morphological family members than Dutch non-identical cognates.

The English and Dutch non-identical cognates and the identical cognates in each version were each matched on English Family Size and Dutch Family Size, English Frequency, and Dutch Frequency, Length, log English Bigram Frequency, and log Dutch Bigram Frequency to 24 English and 24 Dutch non-cognate items, respectively. These non-cognate items only had a noun-reading. **Table 3** presents the characteristics of the cognate and non-cognate stimuli.

**Table 3:** Item characteristics of the experimental items used in Experiment 2.

Stimulus	Length	Log English frequency	Log English family size	Log Dutch frequency	Log Dutch family size
Identical cognates	5.1	3.21	1.93	2.98	1.96
Dutch non-cognate controls	4.9	-	-	3.03	2.43
English non-cognate controls	4.7	3.40	1.74	-	-
English non-identical cognates	4.9	3.56	2.03	3.66	3.07
Dutch non-identical cognates	5.2	3.56	2.03	3.66	3.07
English non-cognate controls	5.0	3.65	1.78	-	-
Dutch non-cognate controls	5.1	-	-	3.51	2.97

The experiment consisted of two item blocks. The proportion of items from each condition was the same in the two parts of the experiment. The presentation order of the items within each item block was randomized for each participant with the restriction that no more than three cognates or non-cognates followed each other directly.

#### Procedure

Participants performed an Dutch-English language decision task. In this language classification task, participants have to decide whether the visually presented stimulus is an existing English or Dutch word by pressing a button corresponding to either the answer ‘English’ or ‘Dutch.’ The study was approved by the ethical committee of the Faculty of Social Sciences at Radboud University (ECG2912-2711-059).

The task was developed and carried out in *Presentation* version 13 (Neurobehavioral Systems^5^) on a HP Compaq Intel Core 2 computer with 1.58 GHz memory and a refresh rate of 120 Hz. Participants were tested individually in a sound proof room. They were seated at a table at a 60 cm distance from the computer screen. The visual stimuli were presented in white capital letters (24 points) in font Arial in the middle of the screen on a dark gray background.

Participants first read the English instructions. These informed them that they would be presented with word strings, and asked them to push the ‘left’ button if the letter string they saw was an existing English word and the ‘right’ button if the letter string was a Dutch word. They were informed that some words in the experiment could belong to both Dutch and English. In those cases, they were free to choose whichever response they liked. They were asked to react as accurately and quickly as possible.

Each trial started with the presentation of a black fixation point ‘+,’ which was displayed in the middle of the screen for 700 ms. After 300 ms the target stimulus was presented. It remained on the screen until the participant responded or until a maximum of 1500 ms passed by. The experiment was divided into two parts of equal length. The first part was preceded by 20 practice trials. After the practice trials, the participant could ask questions before continuing with the test trials. The two parts each contained 84 experimental trials, and each started with three dummy trials.

After completing the language decision task, participants performed the X-LEX (Meara and Milton, 2003). This task was used to obtain a general indication of their proficiency in English in terms of vocabulary knowledge. All participants obtained a score of 3200 or higher, which qualified them as intermediately or highly proficient in English. Finally, participants were asked to fill out a language background questionnaire. The experimental session lasted approximately 18 minutes.

### RESULTS

The data were first screened for high error rates of participants and items. The participant accuracy mean ranged between 90.3 and 100%. Due to the small proportion of errors, data of none of the participants had to be excluded. However, four participants were excluded based on their slow mean RTs (more than 2 SDs from group RT mean) on the task relative to the mean RTs of the other participants.

Items that had more than 20% of errors were removed from the data set. These included two cognate items (*priest* and *thee*) and one non-cognate item (*poem*). Note that responses to identical cognates, which have an identical form in English and Dutch, could never result in errors, because both an English or a Dutch response is appropriate. Incorrect items and null responses were removed from the remaining data set. This resulted in a dataset of 6473 data points. Inspection of the distribution of the response latencies revealed non-normality, with outliers in both tails. An inverse transform (RT = 1000/RT) was most successful in attenuating this non-normality.

As in Experiment 1, the data were analyzed with a linear mixed effects model. We considered the same predictors as in Experiment 1. *Response Language* and *Previous Language* were added as variables. *Response Language* was defined as the value (Dutch or English) of the response given to the preceding word. *Previous Language* corresponded to the language membership of the preceding word (Dutch, English, or in the case of identical cognates, both). Moreover, we added the predictor *Total Family Size* (the sum of the Dutch and English family sizes) to account for possible increased response conflict due to large amount of global activation in the lexicon produced by the family members. The same procedure as in Experiment 1 was applied to obtain the final model.

Both *Dutch Family* Size and *English Family Size* were considered in one model. Both predictors had an inhibitory effect on response latencies when both were included in the same model or when included in a separate model with only one family size measure. Moreover, *Total Family Size* had an inhibitory effect. An ANOVA revealed that the model with *Total Family Size* was slightly better at explaining the variance (as reflected by lower AIC values). Therefore, *Total Family Size* was included in the model in favor of *English Family Size* and *Dutch Family Size*. Further, the predictor *Dutch Frequency* produced an insignificant coefficient and was removed from the model. Finally, *Word Type*, *Identical Cognate,* and *Cognate* were considered. The model with *Identical Cognate* resulted in the best fit of the data.

**FIGURE 2:**
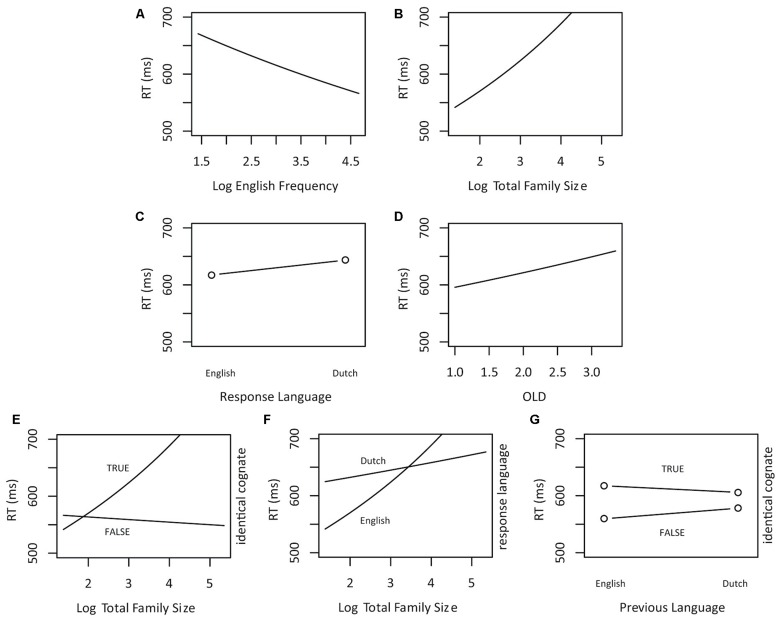
**Partial effects of the significant predictors on response latencies in English-Dutch language decision (Experiment 2). (A)** Log English Frequency, **(B)** Log Total Family Size, **(C)** Response Language (2 levels: English and Dutch), **(D)** OLD, **(E)** the interaction of Log Total Family Size with Identical Cognate (2 levels: True, corresponding to the set of identical cognates, and False, corresponding to the set of non-identical cognates and non-cognates), **(F)** the interaction of Log Total Family size and Response Language (2 levels: English and Dutch), and **(G)** the interaction of Previous Language (2 levels: English and Dutch) and Identical Cognate (2 levels: True, corresponding to the set of identical cognates, and False, corresponding to the set of non-identical cognates and non-cognates).

The final model incorporated two parameters for the random-effects structure of the data: a standard deviation for the random intercept for item (SD = 0.07) and subject (SD = 0.14), as well as a standard deviation for the by-subject random slope for *Trial* (SD = 0.06). The SD for residual error was 0.35. The model contained three numerical predictors (*English Frequency*, *Total Family Size,* and *OLD*), three factorial predictors (*Identical Cognate, Response Language*, and *Previous Language*), and four interactions (*Identical Cognate: Total Family Size*, *Identical Cognate: English Frequency*, *Total Family Size: Response Language*, and *Identical Cognate: Previous Language*). The relevant statistics and corresponding coefficients of the final model are reported in **Table 4**. The significant effects of the final model are visualized in **Figure 2**. In both **Table 4** and **Figures 2E,G**, *Identical Cognate* has two levels: *True* and *False*: the former corresponding to the set of identical cognates, and the latter to the set of non-identical cognates and non-cognates.

**Table 4:** Coefficients of the main effects and interaction effects of the final model, together with the standard error, *t*-values and *p*-values in English-Dutch language decision (Experiment 2).

	Estimate	SE	*t*-value	*p*-value
Intercept	-1.932	0.122	-15.808	0.000
English frequency	-0.085	0.032	-2.659	0.008
Total family size	0.151	0.032	4.697	0.000
Identical cognate *false*	0.109	0.130	0.839	0.369
Response language *Dutch*	0.411	0.085	4.855	0.000
OLD	0.069	0.028	2.511	0.014
Previous language *Dutch*	-0.031	0.024	-1.311	0.181
Total family size: Identical cognate *false*	-0.165	0.038	-4.327	0.000
English frequency: Identical cognate *false*	0.068	0.043	1.592	0.094
Total family size: Response language *Dutch*	-0.120	0.032	-3.800	0.000
Previous Language Dutch: Identical cognate *false*	0.088	0.034	2.615	0.001

A significant facilitatory main effect of *English Frequency* was observed. Further, *Total Family Size* had an inhibitory effect on word processing. Moreover, *OLD* had an overall inhibitory effect, showing that the more distant orthographic neighbors are in terms of orthographic similarity, the harder it is to make a language decision.

The main effect of *Response Language* revealed slower response latencies when Dutch was chosen as response language (including responses to Dutch identical cognates and Dutch non-cognate words). Moreover, we observed an interaction between *Total Family Size* and *Response Language* demonstrating faster RTs for words with a large combined family size when the response language was Dutch.

There was no significant main effect of *Identical Cognate* when multiple interactions were included in the model. *Identical Cognate* interacted significantly with *Total Family Size* and revealed more inhibition with an increasing number of Dutch and English family members for identical cognates than for the other stimuli. Finally, *Identical Cognate* interacted with *Previous Language* showing faster response latencies for non-identical cognates and non-cognates compared to identical cognates when the response language was English.

The possibility of a response strategy was considered in a model predicting the response language chosen by the participant (English or Dutch) on identical cognates only. The same predictors that were considered in the analysis of the complete data set were included. Again, all non-significant predictors were removed.

**FIGURE 3:**
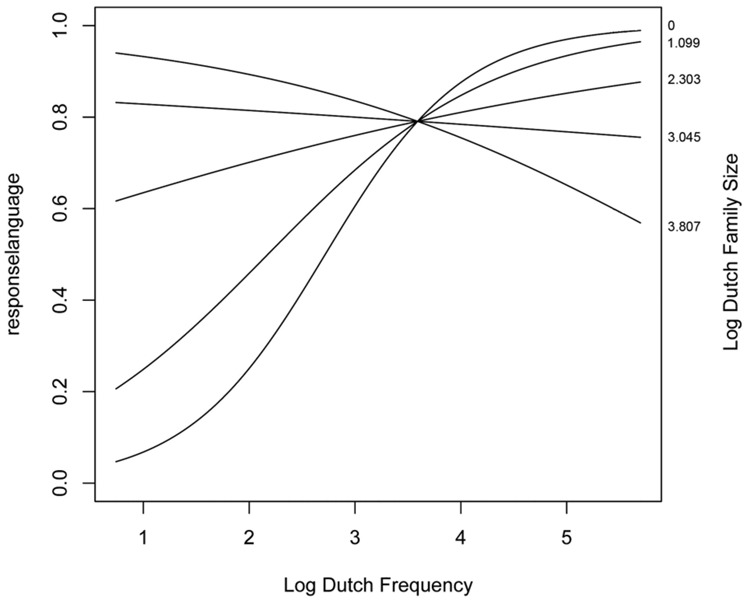
**Significant interaction between Dutch Family Size and Dutch Frequency as a predictor of the choice for response language (0 = English, 1 = Dutch) on identical cognates**.

The final model incorporated two parameters for the random-effects structure of the data: a standard deviation for the random intercept for item (SD = 0.09) and subject (SD = 0.16), as well as a standard deviation for the by-subject random slope for *Trial* (SD = 0.06). The standard deviation for residual error was 0.42. The model contained two numerical predictors (*Dutch Frequency* and *Dutch Family Size*) and one interaction (*Dutch Family Size: Dutch Frequency*). The relevant statistics and corresponding coefficients of the final model are reported in **Table 5**. The significant interaction of the final model is visualized in **Figure 3**.

**Table 5:** Coefficients of the model predicting the choice for response language in identical cognates in Dutch-English language decision (Experiment 2).

	Estimate	SE	*t*-value	*p*-value
Intercept	0.956	0.103	9.270	0.000
Dutch frequency	0.249	0.043	4.840	0.000
Dutch family size	0.249	0.061	4.094	0.000
Dutch family size: Dutch frequency	-0.068	0.018	-3.709	0.000

*Dutch Family Size* interacted significantly with *Dutch Frequency*, revealing that a high *Dutch Frequency* led to more Dutch responses when the *Dutch Family Size* was small (and vice versa). When both the *Dutch Family Size* and *Dutch Frequency* were low, more English responses were given.

**FIGURE 4:**
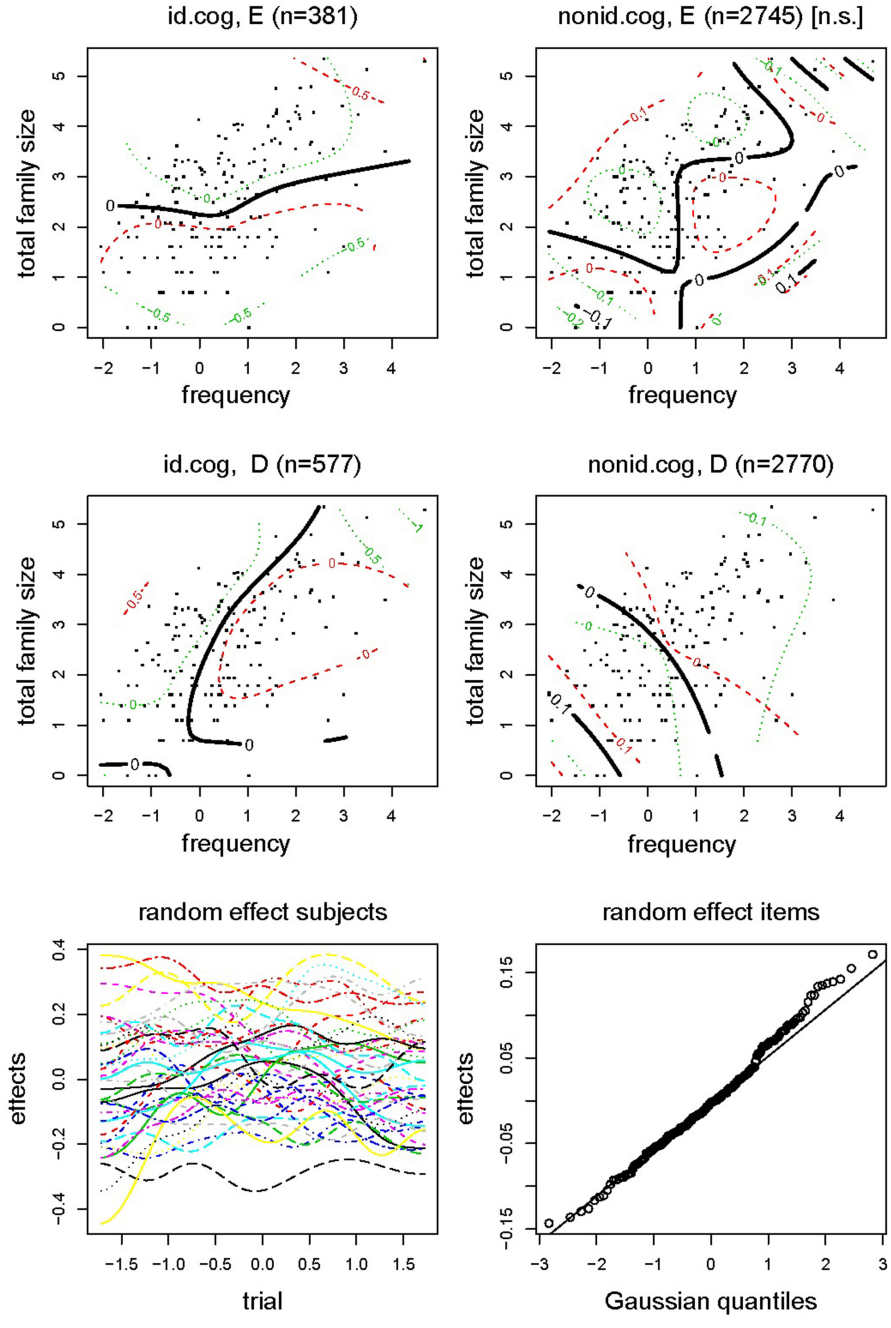
**Experiment 2: GAMM for response latency**.

In order to obtain a more fine-grained picture, we further looked at non-linear relationships involving family size and cognate status. We therefore also analyzed the data by means of a generalized additive mixed model (GAMM)^6^. The parametric part of the model contained the predictor *IRL* specifying the four combinations of *Identical Cognate* and *Response Language*, while the non-parametric part included tensor product smooths for the interactions of *IRL* with *English Frequency* and *Total Family Size,* and smooth terms for item and the interaction of* Trial* by participant. **Table 6** presents the coefficients for the main effects and interaction effects of the GAMM, together with the standard error, *t*-value and *p*-value. **Figure 4** visualizes these effects. The results of the GAMM refined the results of the earlier linear mixed effects model as follows.

**Table 6:** Coefficients of the GAMM predicting response latencies in Dutch-English language decision (Experiment 2).

A. Parametric coefficients	Estimate	SE	*t*-value	*p*-value
Intercept	-1.7157	0.0386	-44.46	0.0000
IRLFALSE.EN	-0.0929	0.0315	-2.95	0.0032
IRLTRUE.NL	0.1603	0.0339	4.73	0.0000
IRLFALSE.NL	-0.0959	0.0321	-2.98	0.0029

**B. Smooth terms**	**Edf**	**Ref.df**	***F*-value**	***p*-value**

Tensor smooth frequency and total family size: IRLTRUE.EN	4.15	4.71	3.28	0.0073
Tensor smooth frequency and total family size: IRLFALSE.EN	6.94	7.65	1.94	0.0532
Tensor smooth frequency and total family size: IRLTRUE.NL	5.26	6.09	2.91	0.0075
Tensor smooth frequency and total family size: IRLFALSE.NL	3.00	3.00	8.37	0.0000
Smooth trial: participant	187.34	368.00	5.04	0.0000
Smooth item	125.154	201.00	1.81	0.0000

In the parametric part of the model, the reference level of *IRL* refers to identical cognates responded to with an English decision (TRUE.EN in **Table 6**). Relative to identical cognates, responses with ‘English’ for non-identical cognates and English non-cognates were faster (by 0.093). Identical cognates that received a ‘Dutch’ response were responded to more slowly than identical cognates receiving an ‘English’ response (by 0.16). ‘Dutch’ responses to non-identical cognates and Dutch non-cognates were faster, just as for English (by 0.096). In other words, identical cognates were difficult to respond to, especially so when participants decided to go for ‘Dutch’ as response.

The non-parametric part of the model showed that for identical cognates responded to with ‘English,’ mainly an effect arose of *Total Family Size*: a greater combined Dutch and English family size slowed the participants’ responses. For identical cognates responded to with ‘Dutch,’ there was mainly a facilitatory effect of *Frequency*. For non-identical cognates and non-cognates in English, *Frequency* and *Total Family Size* were not predictive. Finally, for non-identical cognates and non-cognates with ‘Dutch’ as response language, both *Frequency* and *Total Family Size* were at work. Both effects were now facilitatory. The final two panels of **Figure 4** show a large variability in subjects and items. For subjects, the factor smooths show large differences between fast and slow subjects, plus considerable variation in how they proceeded through the experiment.

**FIGURE 5:**
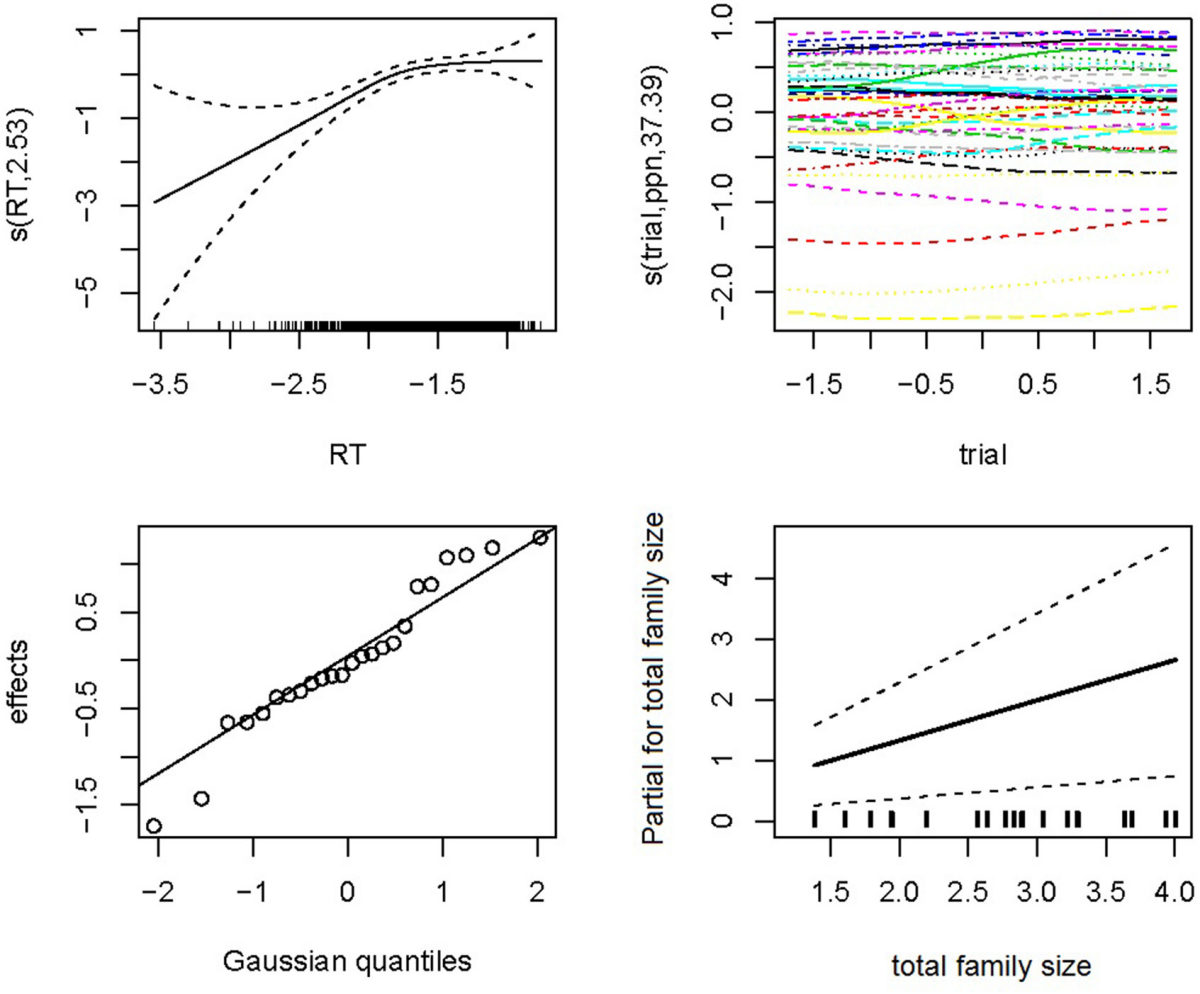
**Experiment 2: logistic GAMM for language choice**.

A second GAMM analysis was performed to analyze the choice for response language upon seeing an identical cognate. The model included the predictor *Total Family Size* as well as smooth terms for *RT*, item, and the interaction of *Trial* by participant. **Table 7** presents the coefficients for the main effects and interaction effects of the model, together with the standard error, *z*-value, and *p*-value. **Figure 5** visualizes these effects that assess the log of the Dutch/English odds ratio. The upper left panel indicates that, as *RT* increases, Dutch is more likely to be selected. For shorter response latencies, however, there is considerable uncertainty about the estimate, suggesting guessing behavior. The upper right panel shows that, with incomplete information about the time series of responses (when only identical cognates are included in the analysis), most of the participant differences concern a language bias on the part of the participants, some preferring Dutch, others preferring English. The lower left panel indicates that the item effects were fairly normal. Finally, the lower right panel presents the effect of *Total Family Size*. The greater the joint English-Dutch family size, the more likely Dutch was as the response category.

**Table 7:** Coefficients of the GAMM predicting the choice for response language in Dutch-English language decision (Experiment 2).

A. Parametric coefficients	Estimate	SE	*z*-value	Pr (>| z| )
Intercept	-1.239	0.654	-1.89	0.0582
Total family size	0.663	0.239	2.78	0.0055

**B. Smooth terms**	**Edf**	**Ref.df**	**Chi-sq**	***p*-value**

Smooth response latency	2.53	3.22	24.0	0.0000
Smooth trial: participant	37.39	368.00	119.5	0.0000
Smooth item	18.34	22.00	89.9	0.0000

In sum, the model on response latencies reveals from the shifts in intercepts, that when dealing with an identical cognate, participants were faster to choose English and slower to choose Dutch. When they chose English, a large *Total Family Size* (mostly coming from Dutch family size) worked against this decision (upper left panel of **Figure 4**). When they chose Dutch, a greater *Frequency* facilitated this response. When dealing with a non-identical cognate or a non-cognate, responses were on average faster: the item’s orthography was informative about the language. For English, lexical distributional properties had no predictivity. For Dutch, *Frequency,* and *Total Family Size* worked in the usual way, both affording facilitation. From the analysis of the language selected for response, we see that participants based their ultimate decision on semantics: the better integrated a word was in the lexical network, as evidenced by a large family size, the more likely a participant was to opt for Dutch. As family sizes in English are probably smaller than those for Dutch for these participants, using family size as a guide to language is a rational choice. Of course, using family size as a rationale for selecting Dutch words must give rise to longer decision latencies when actually a decision is made favoring English. This is exactly what we see in the reaction time data (upper left panel of **Figure 4**).

We conclude that participants performing this language-decision task thus operate under two potentially conflicting sources of information. First, the orthography provides, for non-identical cognates and non-cognates, but distributionally also for identical cognates, a bias toward one or the other language. Second, the semantic activation of a word, gaged by its family size, does not allow a language decision. Participants in this experiment chose to optimize their responses by taking a large family as evidence for their native language. For English, this slowed their responses.

### DISCUSSION

The aim of Experiment 2 was to tap into the task dependency of the family size effect for cognates. In this experiment, we applied a language decision task in which participants had to decide if a visually presented word was either English or Dutch. Because in this task participants have to distinguish the two readings of a word, response conflicts are expected to arise upon seeing a cognate and these conflicts should result in a cognate inhibition effect. We hypothesized that activation of both target and non-target language family members should strengthen the activation of both representations and add to the response competition in cognates.

As was shown in a linear mixed effects model and confirmed by a GAMM, there was a clear dissociation between identical cognates and non-identical cognates in terms of response latencies. Identical cognates were processed more slowly than non-identical cognates and non-cognates, though the main effect of *Identical Cognate* disappeared when multiple interactions with *Identical Cognate* were considered in the linear mixed effects model. The inhibitory effect can be explained as follows. For identical cognates, which have an overlapping similar orthography in both Dutch and English, there is no language specific orthographic cue that will resolve the language decision, and both language responses will be appropriate (participant’s choice). This will induce response competition for identical cognates. The response competition is attenuated in non-identical cognates, because these items contain orthographic cues that resolve the language ambiguity, resulting in no significant inhibition for these types of cognates compared to language specific non-cognates.

The family size effects were found to be inhibitory for both languages (in the final model, both family sizes were combined into one count *Total Family Size*, which resulted in an even larger coefficient for family size). This finding argues against the hypothesis that cross-language family size effects are exclusively driven by the semantic overlap between family members and target word. This would logically always lead to facilitatory effects in cognates. Instead, the inhibitory family size effects observed for both languages show that family size effects are sensitive to task context. Activated family members were found to increase the induced response competition between cognate representations (i.e., the more a word points to both languages, the more difficult it is to make a choice between a Dutch and an English response).

Interestingly, the observed dissociation between identical cognates and non-identical cognates was also reflected in the strength of the combined family size effect. *Total Family Size* interacted with *Identical Cognate*, reflecting a large inhibition effect for identical cognates but not for non-identical cognates and non-cognates. This shows that activation of Dutch and English morphological family members added to the competition in identical cognates, increasing the inhibitory effect for these words.

Surprisingly, although participants were more fluent in Dutch than in English, they were slower when they chose Dutch as a response language (for both items that either require a Dutch response or items that may receive a Dutch response). Moreover, participants were slower on non-identical cognates and non-cognates compared to identical cognates when they were preceded by a Dutch item. This suggests that participants applied a response strategy in which English was set as a default response (cf. the language decision experiment in Dijkstra et al., 2010). Finally, *Total Family Size* moderated the Dutch responses: a Dutch response for words with a large combined family size resulted in faster response latencies.

The possibility of a response strategy was considered in a model predicting the choice for a given response in English or Dutch on identical cognates only. The choice pattern for identical cognates could be predicted from *Dutch Family Size* and *Dutch Frequency*. Identical cognates that were highly frequent in Dutch elicited more Dutch responses than less frequent identical cognates. Similarly, identical cognates that had a high productivity in terms of Dutch family members more often elicited a Dutch response than identical cognates with a smaller number of Dutch family members. However, when both the Dutch frequency and family size were either very low or very high, participants more often pressed the English response button. Relating this finding to the observed pattern in the response latencies, it suggests that our bilingual participants adopted a response strategy in which English was the default response language, which was hindered by the strong Dutch activation. These results were largely confirmed by the GAMM analysis: participants used the combined morphological family (consisting for a substantial part of Dutch family members) as a rationale for selecting Dutch words. This resulted in longer decision latencies when actually a decision was made favoring English

In sum, the language decision results reveal that the direction of the family size effect is sensitive to task-induced processes such as response competition between cognate representations. Furthermore, we found a dependency of the family size effect on the cross-linguistic degree of form overlap in cognates, which is an indication that the activation of family members depends on their similarity to the input word. For instance, the input letter string *work* may activate Dutch a family member like *werkplaats* somewhat less than *hotel* would activate *hotelkamer*, because of the cross-linguistic difference in orthographic overlap between the target words and their family members. In language decision (in contrast to lexical decision), this effect of orthographic overlap becomes visible, because, due to an increased activation of both language nodes for identical cognates, response competition becomes enlarged and magnifies the family size effects.

## GENERAL DISCUSSION

The present study investigated the role of task-dependency and orthographic overlap in activating cross-language family members. By looking at family size effects in cognates, we aimed at answering two main questions. First, is the cross-language family size effect sensitive to language-specific orthographic cues of stimuli, such as the degree of orthographic overlap between cognate representations? Second, is the cross-language family size effect sensitive to more task-dependent processes, such as response competition between cognate representations? These questions were investigated with Dutch-English bilinguals in two behavioral experiments: an English lexical decision task (Experiment 1) and an English-Dutch language decision task (Experiment 2).

In Experiment 1, English lexical decision, a cognate facilitation effect was observed for both identical and non-identical cognates relative to English non-cognates, with the largest effects for identical cognates. Dutch family size was observed to have a facilitatory effect on cognate processing. Further, no interaction between Dutch family size and cognate type was found, indicating that the strength and the direction of the cross-language family size effect did not significantly change as a function of the degree of form overlap in the cognate items.

In Experiment 2, a Dutch-English language decision with the same type of bilinguals as was used in Experiment 1, response competition between Dutch and English cognate representations was experimentally induced by means of a two-choice forced decision about the language membership of the items. Relative to non-cognates, this resulted in an overall inhibitory cognate effect for identical cognates but not for non-identical cognates. English family size had an inhibitory effect on response latencies to both cognates and purely English words. With respect to Dutch family size effects, similar inhibitory effects were observed for cognates and purely Dutch items. Moreover, the inhibitory effects of Dutch and English family size in cognates were stronger when they were combined into one family size count (*Total Family Size*). These results demonstrate that the direction of the within-language and cross-language family size effects (facilitatory or inhibitory) is not only driven by semantic overlap in the morphological family, but is sensitive to other processes that play a role in the task at hand, such as response competition.

Interestingly, the combined family size effect was also found to depend on cognate type: a large combined morphological family induced more inhibition in identical cognates than in non-identical cognates. This can be explained by assuming that identical cognates, due to their complete orthographic overlap, lead to a stronger activation of semantics and of family members than non-identical cognates. In language decision, this complete cross-linguistic overlap might increase the amount of response competition between activated cognate representations.

How do these bilingual family size effects in cognates relate to the findings of earlier and predominantly monolingual studies that argued that the family size effect is a purely semantic effect? We found that the cross-language family size effect is sensitive to the demands posed by the task to be performed. In a task like English lexical decision (Experiment 1), only one language (English) is relevant for responding (“is this an English word or not”), and the activation of English words is assessed against the background activity in the lexicon produced by English non-words. In this task situation, English is not explicitly contrasted with Dutch. Under these circumstances, especially semantic convergence of family members in the two languages seems to determine the direction of the family size effect for cognates, resulting in facilitation. Similar findings arise for generalized lexical decision, in which words of both languages underlie the “yes, it is a word” (e.g., Dijkstra et al., 2005; Mulder et al., 2014). These results are similar to those obtained in the monolingual domain (e.g., Schreuder and Baayen, 1997; De Jong, 2002).

In contrast, in our language decision task (Experiment 2), the two languages must be contrasted explicitly to arrive at a correct response (“is this word English or Dutch?”). Here orthographic language-specific information is relevant for distinguishing activated cognate representations, each of which is linked to a particular response. As a consequence, the processing of cognates suffers from response competition between activated representations. In line with this argumentation, Dijkstra et al. (2010) observed longer response latencies for identical cognates compared to non-identical cognates in language decision. This finding shows that the larger the orthographic overlap in cognates is, the larger the competition between activated representations is as well. Our data attenuate this finding by showing that it is more a complete-incomplete distinction with respect to orthographic overlap rather than a graded effect. In this sense, identical cognates might have a special status that allow for maximal cross-linguistic effects to occur (cf. Mulder et al., 2014).

In fact, in our language decision experiment, semantic convergence between target and family members did not lead to facilitatory effects of family size, even though activated family members are assumed to strengthen the activation of each cognate representation to which they are linked. Due to the response competition between cognates, inhibitory family size effects arose. Especially in identical cognates, a large family size in one of the two languages is not beneficial for word processing in language decision: the activation of a large number of family members that contain language-ambiguous orthographic information (e.g., the activation of *water* in the English family member ***water*** *fall* and Dutch family member *drink* ***water*** for the target cognate *water*) increases the response conflict between competing cognate representations. This results in more inhibition for identical cognates with a large family size in one the two languages relative to non-identical cognates (that contain more language-specific information to resolve the response conflict) with a large family size.

We note that the different direction of the family size effects observed in lexical decision (i.e., facilitation) and language decision (i.e., inhibition) is not due to a difference in the item set, because Mulder et al. (2014) observed also facilitatory effects of family size in lexical decision with an item set that was highly similar to the item set used in our language decision experiment. This indicates that the direction of the effect is not dependent on a specific subset of items but differs as a function of task demands.

Our findings have consequences not only for models explaining morphological effects but also for models of bilingual word processing. According to the MFRM, De Jong et al. (2003), family members are activated through the activated semantic representation of the target to which they are linked, and family size effects occur because of the resonance of activation between the activated family members and the semantic representation.

However, we argue that, in addition to the semantic family effects, in bilingual processing orthographic factors must also play a role. Reconsidering the way in which morphological family members may become activated, two possible routes may be assumed. The first possibility (similar as in the MFRM) is that, upon reading the input *water*, the orthographic representations of ‘water’ in each of the two languages become activated. These may then activate their respective (or shared) semantic representations in each language, which will in turn activate their morphological family members. The second possibility is that family members can also be activated indirectly, via a formal route, e.g., the input *water* activates its family member *water fall*, *drinking water*, etcetera via their orthographic compound representations. Evidence for such bottom–up activation of family members is supported by the early family size effects observed in the ERP study of Mulder et al. (2013). The finding that family size effects occurred around 200 ms after stimulus onset could point at activation via a formal route, as it is not evident that semantic activation already is effective at this point in time. Furthermore, the assumption of a formal route leads to the prediction that there should be family size effects in progressive demasking. Although Schreuder and Baayen (1997) failed to observe family size effects in monolingual progressive demasking, this could have several causes. For instance, orthographic factors on family size effects might only play a role in bilingual processing, because of competing representations of different languages. Alternatively, the traditional ANOVA in their paper might not have been sensitive enough to pick up family size effects in progressive demasking. A replication of this monolingual study and additional bilingual progressive demasking experiments might unravel under which conditions the formal route plays a role.

In sum, the data presented in this paper support an account proposing two routes of activation for family members depending on the task at hand: a direct, bottom–up route via the orthographic representation of the target and an indirect, semantic route via resonance with the target. The explanation of family size effects presented above thus proposes a bilingual extension of the MFRM model of De Jong et al. (2003) in terms of adding an orthographic route to activate family members. Importantly, resonance of activation between the semantic level and lemma level can still occur via this route, and in many task situations, the semantic route may be the dominant route.

**FIGURE 6:**
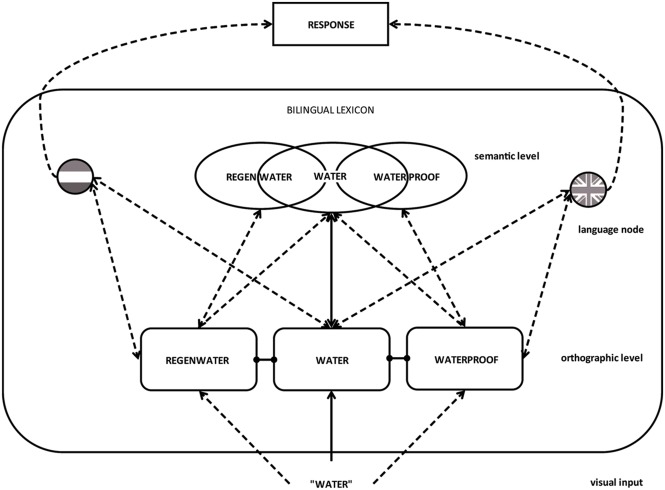
**Schematic representation of activation of family members within a bilingual interactive activation model based on BIA+.** The activation of the morphological family of a target word can affect the processing of a target word *positively* when (a) family members are activated via a semantic route, or (b) family members are activated via an orthographic route but there is resonance of activation between semantics and orthography, and *negatively* when (a) activated family members map onto a different response or (b) family members are activated via an orthographic route and resonance of activation between semantics and orthography is still under development.

Our data are also in line with language non-selective access accounts of bilingual word processing, such as in bilingual interactive activation models like the BIA+ model (Dijkstra and Van Heuven, 2002). Although it allows co-activation of orthographically or phonologically related lexical items, the BIA+ model has no specific account for resonance between family members and the target to which they are linked. Integrating the MFRM model of De Jong et al. (2003) within the BIA+ model would result in a model that allows activation of family members via an orthographic route and a semantic route, and allows resonance between semantic and orthographic representations. This model is displayed in **Figure 6**^7^.

However, a further model extension is required to account for all our bilingual data. In a task situation in which two languages need to be distinguished, such as language decision, activation of language membership information determines the role of the activated family size. In language decision, a response conflict arises when activated representations from two languages overlap in form (e.g., cognates or interlingual homographs) and are linked to different responses. The response competition is more directly dependent on language membership information than on semantic convergence between target and family members. Inhibitory effects of family size of both languages can be explained by summed language membership activation that increases the response conflict. Language membership information based on the orthographic input should come available in parallel to the semantic representation that has been activated (cf. Van Kesteren et al., 2012). However, additional effects of response competition might influence later stages of word processing also when family members have been activated via the overlapping semantic representation. The effect of summed language membership activation on response competition is weaker when the orthographic overlap between the target word and family members is reduced (i.e., there is less activation sent to the inappropriate language membership node). Thus, in an interactive activation account, family size effects can ultimately be explained via three mechanisms: facilitation due to *orthographic* co-activation of morphological family members in cognates, facilitation due to *semantic* co-activation in cognates, and *response inhibition* due to co-activated morphological family members, captured in one value as summed language membership activation (as in language decision)^8^.

In sum, we observed effects of cross-language family size for cognates in two paradigms (English lexical decision and English-Dutch language decision) that have similarities and differences in the demands they make on the participant. Semantic resonance between family members and target word was shown to be a major mechanism underlying family size effects, but orthographic overlap also played a role when it was relevant for making the correct response in language decision. All in all, we argue that the effect of morphological family size is sensitive to both semantic and orthographic factors, and also depends on task demands. As such, the research in this paper is of fundamental importance to the study of morphology, because it clarifies how simplex words activate morphologically complex associates (their family members) in bilingual word processing.

## Conflict of Interest Statement

The authors declare that the research was conducted in the absence of any commercial or financial relationships that could be construed as a potential conflict of interest.

## Footnotes

1 Lemmas are abstract word units. In Schreuder and Baayen’s (1995) model of morphological processing, lemma nodes links form information at the access level with higher-order semantic and syntactic information. See also Taft (2011), who discussed an interactive activation framework incorporating a lemma level that captures lexical information.

2 Recently, Mulder et al. (2014) investigated English primary and secondary family size effects in English visual lexical decision with Dutch(L1)-English(L2) bilinguals. Their stimulus materials included both Dutch-English cognates and purely English items. No effects of Dutch primary and secondary family size effects were observed on the set of cognates. The authors argued that this occurred because the English family size was varied and, consequently, took away part of the effect. However, they hypothesized that cross-language family size effects might be observed in a design in which the family size of the target language is controlled for. This design is adopted in the present study.

3 http://www.nbs.com

4 The materials were highly similar to the materials used in Mulder et al. (2014). All 24 identical cognates, 24 out of 25 English non-identical cognates and a large portion of the control words used in Mulder et al. (2014) were also used in Experiment 2. In this study, the family sizes of Dutch and English were varied.

5 http://www.nbs.com

6 A generalized additive mixed model (GAMM) extends the general linear model by allowing non-linear relationships between one or more predictors and the dependent variable. It consists of a parametric part that is identical to that of a standard (generalized) linear model, and a non-parametric part that provides functions for modeling non-linear functional relations in two or higher dimensions. GAMMs are especially useful for modeling interactions of numerical predictors. Whereas multiplicative interactions in the generalized linear model impose a very specific (and highly restricted) functional form, the so-called tensor product smooths of GAMMs make it possible to fit wiggly regression surfaces and hypersurfaces (see Wood, 2006, for further details).

7 Note that, in contrast to Schreuder and Baayen (1995) and De Jong et al. (2003), the BIA+ model does not specify a level for lemmas and morphemes, but contains an orthographic level.

8 See Mulder et al. (2014) for a further discussion on how semantic and form similarity between a target word and (more distant) family members affect target word processing.
